# Cutaneous Laser Therapy for Residual Limb Skin Pathology: A Narrative Review Bridging Dermatologic Laser Research With Clinical Perspectives and Considerations for Multidisciplinary Care Teams

**DOI:** 10.7759/cureus.99996

**Published:** 2025-12-24

**Authors:** Ford M Lannan, Alexander J Nardone, Rachel C Ruda, Tawnee L Sparling, Colin J Harrington, Sunghun Cho, Douglas G Smith

**Affiliations:** 1 Dermatology, Landstuhl Regional Medical Center, Landstuhl, DEU; 2 Medical Education, Naval Medical Center San Diego, San Diego, USA; 3 Dermatology, Uniformed Services University of the Health Sciences, Bethesda, USA; 4 Physical Medicine and Rehabilitation, Uniformed Services University of the Health Sciences, Bethesda, USA; 5 Orthopaedics, Walter Reed National Military Medical Center, Bethesda, USA; 6 Orthopaedics, University of Washington School of Medicine, Seattle, USA

**Keywords:** cutaneous laser, laser therapy, major amputation, person with limb loss, prosthetic rehabilitation, prosthetics, residual limb, scar treatment, traumatic limb loss

## Abstract

Cutaneous laser therapies are widely employed in dermatology for hair removal, scar remodeling, and skin rejuvenation, yet their use in managing skin conditions specific to residual limbs after amputation remains underexplored. Individuals with limb loss frequently experience skin pathology such as folliculitis, hyperkeratosis, scar contracture, chronic wounds, and volume instability, which can significantly impair prosthetic fit, function, and quality of life. This narrative review synthesizes existing dermatologic laser applications and clinical experience to explore their potential adaptation for residual limb care. Cutaneous lasers offer minimally invasive options with favorable safety profiles to address chronic wounds, contracted scars, inflammatory skin disorders, and follicular pathologies common in this population. Treatment benefits may include improved scar remodeling, enhanced wound healing, reduction of contractures, and facilitation of medication delivery, all contributing to better prosthetic compatibility and patient outcomes. While side effects such as pain and postinflammatory hyperpigmentation can occur, laser procedures are generally well tolerated. Despite a growing population of persons with amputation in the United States and an increasing incidence due to advances in trauma care, laser therapy remains an underutilized tool in the multidisciplinary rehabilitation of persons with limb loss. This review highlights the clinical rationale, therapeutic potential, practical considerations, and precautions relevant to laser use on residual limbs, emphasizing the need for greater awareness among healthcare providers. Given the limited direct research in this field, extrapolation from broader dermatologic literature supports laser therapy as a promising adjunct to improve skin health and function in persons with amputation. Further studies are warranted to establish standardized protocols and optimize outcomes specific to residual limbs.

## Introduction and background

There are over two million persons with amputation living in the United States, a number expected to increase to over three million in the coming years [1]. This small subset of the population is faced with high degrees of morbidity as a result of their limb loss [2], and oftentimes depends on the use of a prosthesis for mobility, function, and improved quality of life [3]. In the military, technological advances in armor and equipment, combat first aid, and evacuation capabilities have resulted in decreased mortality and an increase in nonlethal injuries, particularly involving the extremities. Traumatic limb loss in this population can severely affect an individual’s quality of life and functional status [4].

The optimization of residual limb health is essential in the limb loss population, as a significant number of complications are dermatologic in nature and frequently lead to residual limb pain and functional impairment [5-9]. Such dermatoses may occur due to a combination of shear and stress forces, increased moisture and humidity, and extensive exposure to the chemical compounds of the prosthesis [5]. Furthermore, amputations disrupt neurovascular structures and alter lymphatic drainage, leading to local immune dysregulation and predisposition to the occurrence of disease [10,11].

The most common skin diseases at amputation sites include irritant and contact dermatitis, epidermal inclusion cysts (EICs), erosions and ulcers, and bacterial infections such as folliculitis. Other less common conditions include psoriasis, fungal infections such as tinea and candida, malignancies, and verrucous hyperplasia [7,11-14]. The reported prevalence of these diseases in persons with amputation varies widely, ranging from 16% to 74% [13-18]. Skin disease has a significant negative impact on the physical and mental health of persons with amputation and can lead to impaired prosthetic function, comfort, and quality of life [11,19].

Laser therapy offers a safe and effective form of prophylactic and therapeutic treatment for many dermatologic disorders in individuals with limb loss [9,20]. This narrative review presents the available literature on laser-based treatments for dermatologic pathology on residual limbs. As there is a dearth of studies specific to this population, this review also includes literature on conditions not specific to residual limbs where the therapeutic approach is considered generalizable. The goal is to provide a consolidated overview of common laser applications and devices to guide clinical practice and future research.

## Review

Anatomy of the skin

**Figure 1:**
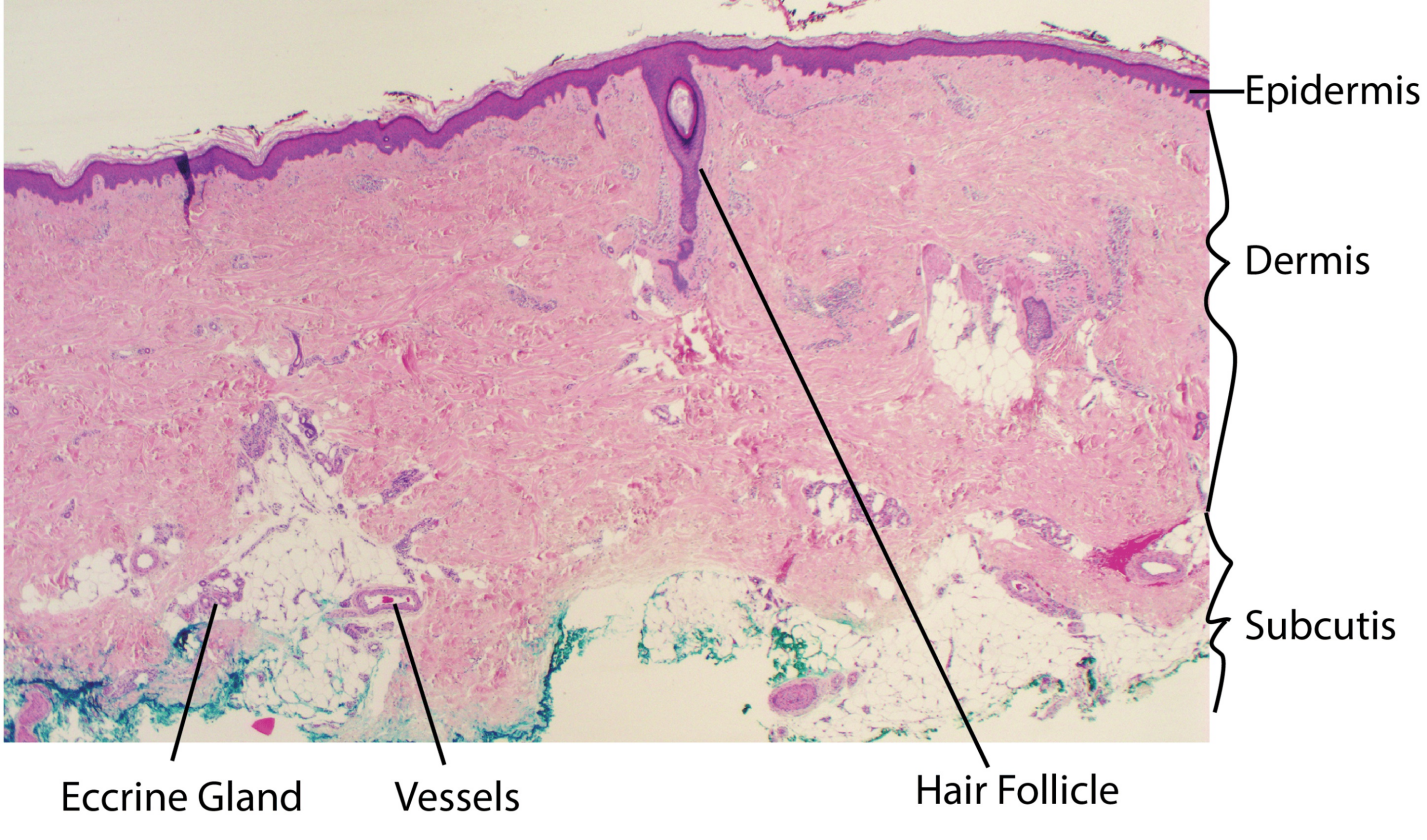
Anatomy of the skin Micrograph of normal human skin of the leg stained with hematoxylin and eosin stain at 20x power. Solid lines denote important anatomical structures. Image Credit: Authors; the patient provided written informed consent for the use of this image in this publication.

The skin and its accessory structures form the integumentary system of the body and are composed of three layers: the epidermis, the dermis, and subcutaneous fat (Figure 1). The epidermis is the avascular superficial layer of the skin. It is further divided into four layers from deep to superficial: the stratum basale, stratum spinosum, stratum granulosum, and stratum corneum. A fifth layer, the stratum lucidum, is found between the stratum granulosum and stratum corneum in thick skin over the palms and soles and is typically not present within residual limb skin. The stratum basale contains the basal stem cells that will become keratinocytes, Merkel cells that relay touch to afferent nerve fibers, and melanocytes that produce melanin to give skin its pigment. The more superficial layers of the skin are composed of keratinocytes that produce keratin and mature into a protective barrier of stratified squamous epithelium. Keratinocytes are interspersed with dendritic cells (Langerhans cells) that help clean up debris and phagocytose pathogens [21].

The dermis is a thick layer of connective tissue that contains the blood vessels, lymphatics, nerves, and adnexal structures of the skin. Fibroblasts in the dermis produce elastin, which gives skin its pliable and elastic qualities, and collagen, which penetrates into the subcutis and gives skin its tensile strength and structure [21]. Eccrine glands have tubular ducts that extend through the epidermis and excrete sweat to thermoregulate the body. Hair follicles produce hair shafts, but also house epithelial stem cells in the dermis that can migrate superiorly to help with wound healing in partial thickness injuries to the skin [22].

The subcutis, also known as the hypodermis, consists of well-vascularized loose areolar connective tissue and adipose tissue. It functions as an insulator, shock absorber, energy reserve, and connection to the underlying fascia of muscle and bone [21].

Basic theory of lasers

**Figure 2:**
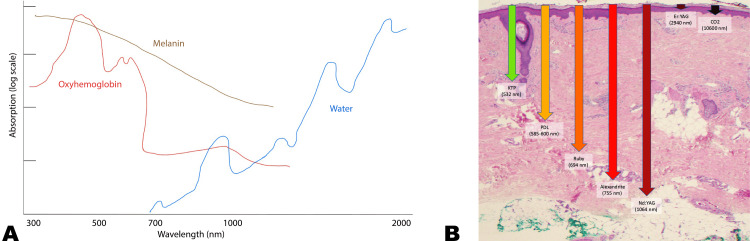
Absorption spectra of target chromophores and depth of penetration by common cutaneous lasers. (A) Absorption spectra. The different absorption spectra of target chromophores allow clinicians to select different lasers for a desired effect. (B) Depth of penetration by various lasers. Please note that the penetration denoted here does not necessarily limit the total depth of penetration, i.e. fractional erbium-doped yttrium aluminum garnet (Er:YAG) and carbon dioxide (CO_2_) lasers can vaporize channels through the epidermis and deeper into the dermis. Image Credit: Authors; for (B), the patient provided written informed consent for the use of this image in this publication.

Lasers exert their effect on the skin based on the principle of selective photothermolysis, which aims to heat up and destroy a specific target (chromophore). By balancing multiple factors such as wavelength, pulse duration, spot size, and fluence, thermal injury is localized to the target, minimizing harm to surrounding tissue. The wavelength, usually a fixed constant in a laser device, is preferentially absorbed by one or more target chromophores of the skin: melanin, hemoglobin, and water. Each of these chromophores has a specific absorption spectrum (Figure 2A). Penetration through the skin is also dependent on wavelength. Longer wavelengths will reach deeper planes, up to about 1200 nm, when absorption by water begins to dominate and decreases penetration (Figure 2B). To confine the heat from the laser, the pulse duration or exposure time must be less than or equal to the target's thermal relaxation time (TRT), its time to cool by 50%. Additionally, the spot size, or beam diameter, affects penetration, with larger spots reaching deeper structures. Finally, the fluence represents the energy density, which controls the strength of the laser beam [23].

​​​​Further care is taken to avoid damage to the skin surrounding a target tissue. Damage to the epidermis due to absorption of energy by melanin can be mitigated by utilizing longer wavelength lasers when possible and cooling the skin prior to, during, and after laser therapy. For lasers with longer pulse durations (>5 milliseconds), concomitant cooling, known as parallel cooling, can be performed by contacting a cold sapphire window with the skin and is useful for those longer durations. Other methods of cooling the epidermis include cold air, cold gels, and precooling of the skin with cryogen spray, which is useful for shorter pulse durations [23].

General considerations for practitioners

Although physiatrists and other non-dermatologic providers may not directly administer laser therapy, understanding its general safety considerations remains essential for the broader multidisciplinary team involved in the care of persons with limb loss. This knowledge supports appropriate referrals, enhances patient counseling, and ensures effective peri-procedural coordination in collaborative clinical settings.

A thorough medical history is critical to determine a patient’s suitability for laser therapy. Postprocedural wound healing may be significantly impaired in patients with autoimmune connective tissue diseases, diabetes mellitus, or a history of tobacco use [23]. While some lasers have been used to treat autoimmune dermatologic conditions such as vitiligo and psoriasis, other lasers can exacerbate existing lesions or trigger new lesions via the trauma-induced Koebner phenomenon [24]. Carefully selected laser settings are key to minimizing these adverse outcomes.

Personal protective equipment for both the patient and practitioner is essential. Ocular injury was among the first recognized hazards of laser use, described in Goldman’s 1963 landmark paper on dermatologic laser applications [25]. Melanin and hemoglobin, both present in ocular structures, can absorb laser energy, leading to serious complications such as retinal and iris burns, hemorrhage, or even perforation [23,25]. Blindness may occur rapidly and painlessly, even from as little as 1% of beam reflection off a shiny surface. Laser safety eyewear must be specific to the laser’s wavelength and rated with an appropriate optical density (OD), typically ≥4 for dermatologic use, as printed on the eyewear. Additionally, opaque eye shields should be used for patients whenever possible, especially when treating near the eyes [23].

Lasers also pose a fire risk, especially in oxygen-enriched environments, as they can ignite flammable items such as hair, drapes, and clothing. CO₂ and erbium-doped yttrium aluminium garnet (Er:YAG) lasers, commonly used for ablative procedures, pose the highest risk due to their affinity for water, allowing them to heat a wide range of materials. Practitioners should place lasers in standby mode when not in use to avoid accidental foot switch activation. Hair near the treatment site should be moistened, and flammable prepping solutions should be allowed to dry before treatment. Intraoperative oxygen levels should be kept below 40%, and fire safety tools, such as a water bucket and fire extinguisher, should be readily available [23].

Finally, laser use can also generate surgical smoke, especially when treating hair-bearing areas. A smoke evacuator and a high-filtration mask (sub-micrometer capacity) are recommended to protect both the patient and the provider [23].

General considerations for patients

Laser treatments are often described as feeling like a rubber band snapping against the skin. In our experience, most procedures are well tolerated and performed in outpatient settings without sedation. For operative or post-procedural pain, management may include topical anesthetics, forced air cooling, or contact cooling. For more extensive treatments, or in patients with specific needs like hyperesthesia or post-traumatic stress disorder, additional options like nerve blocks, oral analgesics, or, rarely, conscious sedation or general anesthesia may be appropriate [23].

Among patients with acquired limb loss, a common concern is the potential disruption in prosthesis use, as recovery timelines vary by laser modality. Post-procedural care is not necessary for the majority of patients who undergo nonablative laser procedures, as there is minimal erythema and edema to the treated areas [26]. Patients are encouraged to resume normal activities, including prosthesis use, post-procedure. In the rare instance of a robust inflammatory reaction during or following the procedure, which may manifest as significant pain, erythema, edema, or even blistering, ice is applied to the affected area followed by a high-potency topical corticosteroid, such as clobetasol ointment [27]. We have the patient continue the post-procedural application of ice and topical corticosteroid to be applied in the evening before bed to mitigate the acute inflammatory response. Topical petrolatum can be used liberally starting the following day until re-epithelialization.

Ablative procedures require more caution due to the need for re-epithelialization. Maintaining an intact skin barrier is critical for preventing complications. Patients may usually shower within 24-48 hours post procedure, though submerging the residual limb should be avoided for at least 72 hours. Post procedure, a thin layer of petrolatum is applied to the treated area, and patients are instructed to continue until re-epithelialization of the skin. It is also recommended to avoid weight-bearing and prosthesis use for 48-72 hours or until complete re-epithelialization occurs. Accordingly, patients undergoing lower extremity treatment are advised to bring a wheelchair or crutches to the appointment. After 72 hours, patients may typically return to full activity. Compression and cold therapy during the initial 24-48 hours can help reduce inflammation and discomfort [27].

Patients should be counseled about the risk of post-inflammatory hyperpigmentation (PIH), a cosmetic side effect that occurs when melanin is released following damage to the stratum basale and is then taken up by macrophages, clearing cellular debris. Additionally, inflammatory mediators like prostaglandins and leukotrienes can stimulate melanocyte activity, leading to increased melanin production and darker pigmentation [28]. PIH is more likely to occur in individuals with darker skin tones, likely due to higher epidermal melanin, more reactive melanocytes, and larger melanosomes [29]. Although PIH can be distressing, anticipatory guidance is often sufficient to address concerns, particularly in patients with limb loss. In most cases, PIH fades over time and rarely becomes a significant issue, even in patients with darker skin tones. 

Patients with post-traumatic stress disorder (PTSD) require special consideration when undergoing laser therapy, as the procedure's sensory stimuli, visual, auditory, and olfactory, may act as trauma triggers, provoking distress or re-experience of symptoms [30]. Hypervigilance, a core symptom of PTSD, may be exacerbated by opaque eye shields, as losing visual awareness of their environment can heighten feelings of vulnerability or anxiety [31]. When clinically appropriate, laser-rated protective goggles that permit vision should be offered as an alternative to fully occlusive eye protection, except when treating periorbital areas.

Additionally, certain lasers, such as the Er:YAG, emit sharp, repetitive acoustic pulses, which can induce the exaggerated startle response characteristic of PTSD [32]. Similarly, olfactory triggers, like the odors produced during laser ablation or hair removal, may also be highly evocative and distressing. To mitigate these effects, a test spot can be performed before initiating a full treatment session to assess patient tolerance. Auditory distress can be reduced with the use of foam earplugs or noise-canceling headphones playing patient-selected music. Olfactory triggers may be addressed with smoke evacuators, and for patients particularly sensitive to the smell, a small amount of essential oil may be applied just below the nasal aperture on a cotton-tipped applicator to mask the odor. These anticipatory adjustments can significantly improve patient comfort, procedural tolerance, and overall treatment experience.

Types of lasers

A variety of laser platforms are employed to treat the dermatologic conditions that affect individuals with limb loss. The selection of an appropriate treatment strategy depends on factors such as scar maturity, skin integrity, tissue compliance, and individual functional goals. While numerous laser types exist, those outside the scope of treating the residual limb are beyond the focus of this paper. 

Vascular Lasers

Vascular lasers are primarily used to treat the erythematous and vascular components of hypertrophic scars. The pulsed dye laser (PDL) (585-600 nm) is the most frequently used modality, targeting hemoglobin to induce selective coagulation and subsequent fibrosis of microvasculature. A common side effect is post-treatment purpura, which typically resolves with time. The potassium titanyl phosphate (KTP) (532 nm) laser is another effective option and is associated with a lower incidence of purpura [23].

In addition to vascular targeting, these lasers contribute to scar remodeling through bulk dermal heating, which stimulates fibroblast activity and promotes collagen remodeling [23]. This mechanism helps soften hypertrophic scars and improve texture and pliability, even in cases where erythema is not the dominant feature.

Caution is warranted when treating patients with darker skin phototypes, as shorter wavelength lasers may also be absorbed by melanin, increasing the risk of epidermal damage, blistering, and PIH [33-35]. For these patients, lower fluences or longer-wavelength lasers, such as the 1064 nm neodymium-doped yttrium aluminum garnet (Nd:YAG), are often preferred due to their deeper penetration and reduced melanin absorption.

Nonablative Fractional Resurfacing (NAFR)

NAFR lasers improve scar texture and pigmentation without disrupting the epidermal barrier. Devices such as the 1320 nm Nd:YAG, 1450 nm diode, 1540 nm erbium:glass, and 1927 nm thulium use water as a chromophore to generate dermal heating and collagen remodeling while minimizing downtime [23,36]. These lasers are well tolerated, safe for outpatient use, and suitable for patients with a variety of skin types.

Ablative Fractional Resurfacing (AFR)

AFR lasers create controlled micro-injuries that ablate columns of tissue within the epidermis and dermis, stimulating significant remodeling while preserving surrounding skin to facilitate healing. CO₂ (10,600 nm) and Er:YAG (2940 nm) lasers are used for this purpose, with Er:YAG offering reduced collateral thermal damage [23,36]. AFR is more effective than NAFR for improving scar thickness and texture, but it also carries a higher risk of complications, including delayed healing, PIH, and scarring, particularly at higher treatment densities and in patients with darker skin [37]. Importantly, AFR is typically deferred until scars have matured, as premature treatment of immature scars may worsen inflammation and fibrosis.

Fully Ablative Lasers

Fully ablative lasers treat the entire skin surface in a single pass, producing more dramatic resurfacing outcomes. However, they are associated with increased thermal injury, prolonged recovery, and pigmentary alterations. Since the development of fractional technology, these lasers are now used less frequently due to their higher risk profiles and longer downtime [23].

Indications and considerations for treatment

Specific clinical indications for laser therapy depend on the context of their impact on rehabilitation and long-term prosthetic use in the limb loss population. Key considerations for treatment, including timing, patient selection, and tissue response to laser energy, are highlighted and summarized in Table 1. 

**Table 1:** Indications for laser therapy in dermatologic conditions of the residual limb 5-FU: 5-fluorouracil; CO_2_: carbon dioxide; Er:YAG: erbium-doped yttrium aluminum garnet; AFR: ablative fractional resurfacing; NAFR: non-ablative fractional resurfacing; Nd:YAG: neodymium-doped yttrium aluminium garnet; PDL: pulsed dye laser; PLLA: poly-L-lactic acid

Indication	Pathophysiology	Laser Modality	Rationale	Key Considerations
Delayed wound healing	Mechanical shear, pressure, and chronic inflammation compromise vascular supply and tissue repair in the postoperative period.	AFR (CO₂, Er:YAG)	Photobiomodulation enhances angiogenesis, fibroblast proliferation, and collagen remodeling.	Use low fluence; consider early intervention for chronic wounds unresponsive to conservative care.
Chronic ulceration	Repetitive shear and poor socket fit cause late-onset ulcers even after initial wound closure	AFR (CO₂, Er:YAG)	Selective ablation of fibrotic tissue with stimulation of neocollagenesis	Minimally invasive alternative to surgical debridement
Atrophic scars	Loss of dermal collagen and subcutaneous fat reduce tensile strength, leading to increased risk of injury from prosthesis use	AFR (CO₂, Er:YAG)	Stimulates neocollagenesis and dermal remodeling to restore pliability and resilience.	Use low-density, low-energy settings to avoid thermal injury in fragile skin.
Hypertrophic scars	Excess collagen deposition and vascular proliferation cause raised, painful, pruritic lesions limiting prosthetic fit and physical rehabilitation	Fractional lasers (AFR, NAFR); vascular lasers (PDL, Nd:YAG)	PDL and Nd:YAG reduce erythema and inflammation; AFR, NAFR promote collagen remodeling and reduce contractures	Treat mature scars with AFR; treat immature scars with lower density NAFR or vascular lasers
Laser-assisted drug delivery (LADD)	Thickened scars limit penetration of topical corticosteroids or antifibrotic agents.	AFR (CO₂ or Er:YAG) followed by topical corticosteroid, 5-FU, or PLLA	Microchannels enhance transdermal absorption and therapeutic efficacy.	Increased drug delivery increases the risk of steroid induced adverse effects
Laser hair removal	Hair growth at the prosthetic interface cause friction, pain, and recurrent inflammation	Alexandrite or long-pulsed Nd:YAG	Selective photothermolysis of hair follicles reduces mechanical friction and subsequent inflammation.	Use wavelength appropriate for skin phototype; may require maintenance treatments.
Epidermal inclusion cysts	Shear-induced keratin invagination causes cystic lesions that impair prosthetic tolerance.	AFR (CO₂ or Er:YAG) or long-pulsed Nd:YAG laser fenestration	Precise ablation removes cyst wall while minimizing collateral tissue damage.	Best for small, non-infected cysts; reduces scarring risk compared to surgical excision
Psoriasis (localized)	Koebnerization and chronic mechanical trauma trigger localized psoriatic plaques on residual limb.	Excimer	Induces apoptosis of keratinocytes and T lymphocytes leading to reduced inflammation.	Use for localized refractory plaques; avoids steroid-associated adverse effects.

Wound Healing Following Amputation

Wound healing in the early postoperative period after amputation is often compromised by both mechanical and biological factors. The residual limb is not biomechanically optimized for weight bearing and prosthetic loading, particularly in the immediate postoperative period, which complicates soft tissue healing [12]. Mechanical pressure over the surgical scar can lead to wound dehiscence and compress adjacent capillary networks, thereby reducing oxygenation, cell migration, and nutrient delivery-key elements of successful healing [8]. The use of socket-based prostheses introduces friction and shear forces that frequently result in atrophic scarring at the skin-socket interface. This tissue is characterized by a thinned epidermis and compromised vascular supply, making it highly friable and mechanically intolerant [38].

A study by Dillingham et al. reported that fewer than half of patients with lower extremity trauma-related amputations were satisfied with their prosthetic use. Notably, nearly 25% of these patients experienced delayed wound healing, skin irritation, or persistent pain in the early postoperative and rehabilitation phases [39]. Hypertrophic scarring and uneven skin texture from both surgical trauma and prosthetic use further impair prosthetic fit, exacerbate discomfort, and hinder participation in physical therapy [12,40].

In addition to mechanical stress, the wound environment in people with amputation is often characterized by prolonged inflammation. The presence of bacterial biofilms is known to disrupt normal wound healing by promoting protease activity that degrades extracellular matrix components and growth factors necessary for collagen deposition and tissue repair [40,41]. Traumatic amputations may leave behind foreign material, bone fragments, or induce subcutaneous heterotopic ossification, all of which sustain the inflammatory process by overwhelming local macrophage clearance mechanisms [4,8,42]. These factors together contribute to the development of chronic, non-healing wounds shortly after amputation surgery.

In the setting of a chronic, non-healing wound that did not respond to conventional wound care, Schumacher et al. demonstrated the use of AFR with a CO₂ laser as a method of “photomicrodebridement” [40]. In their case report, a 22-year-old male with bilateral transfemoral amputations developed a persistent ulceration on the left residual limb following a blast injury. Despite standard treatments, including wound care and silver nitrate, healing failed until the patient received a single AFR session. Within three weeks, the wound showed improved healing, pliability, and durability, allowing resumption of prosthetic rehabilitation. While this report focused on a wound occurring in the late post-rehabilitation phase, the observed outcomes suggest that AFR may have utility for similarly persistent wounds in the early rehabilitation phase, particularly in cases where prosthesis adjustments do not result in clinical improvement [40].

Late-Onset Ulceration in Active Prosthesis Users

Even after initial wound closure and successful rehabilitation, individuals with limb loss, particularly those who are physically active, are at risk for developing ulcerations over the residual limb. These late-onset wounds can emerge months to years after the initial amputation and are often linked to chronic mechanical loading from high-frequency prosthetic use [16]. Repeated shear forces, poor socket fit, residual limb volume fluctuation, and compromised skin integrity all contribute to tissue breakdown over time. These ulcerations are especially concerning as they can interrupt mobility, limit independence, and lead to further complications such as infection or reoperation.

Conventional management of these late-stage wounds has relied heavily on surgical debridement to remove necrotic and poorly vascularized tissue. However, surgical debridement is invasive and necessitates sedation, operative facilities, and recovery periods that delay prosthetic use and disrupt daily life [40]. As an alternative, AFR has shown promise in managing chronic ulcerations through a minimally invasive outpatient approach. Compared to surgical debridement, AFR offers significant advantages: it minimizes downtime, avoids the risks of general anesthesia, and allows for precise removal of fibrotic or necrotic tissue while simultaneously stimulating dermal remodeling through fibroblast activation and collagen regeneration [40].

Atrophic Scars

**Figure 3:**
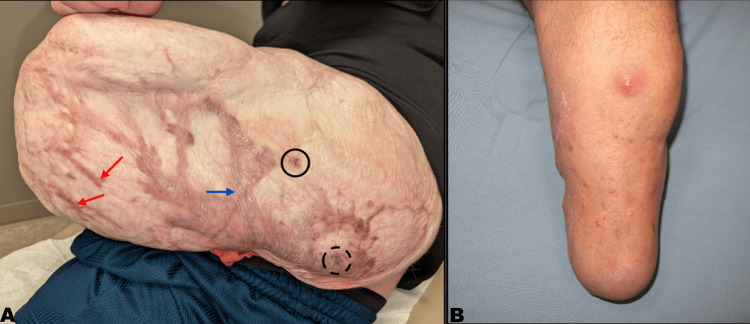
Complex Scar and Folliculitis (A) Complex scar photograph. Red arrows point to areas of hypertrophic scar with surrounding erythema. Blue arrow shows a scar with hypopigmentation and “cigarette paper” atrophy of the epidermis. Solid circle indicates area of hyperpigmentation and dotted circle indicates area of traumatic tattooing. (B) Folliculitis, with erythematous pustules and papules, can lead to a deeper infection such as a furuncle. Image Source: Authors; the patients provided written informed consent for the use of these images in this publication.

Atrophic scars result from the loss of dermal collagen following traumatic or inflammatory injury to the skin (Figure 3A) [36]. The underlying pathophysiology involves collagenase- and metalloprotease-mediated degradation of connective tissue, facilitating angiogenesis but undermining dermal support and subcutaneous fat. These processes are exacerbated when inflammation is prolonged or when deeper soft tissues are disrupted [43]. In patients with amputations, atrophic scars on the residual limb are especially problematic due to their reduced dermal thickness and compromised tensile strength, increasing the risk of mechanical injury during prosthetic use.

Laser therapy for atrophic scars focuses on stimulating neocollagenesis to restore skin texture and resilience. AFR with CO₂ or Er:YAG lasers is the primary modality used, though it must be applied cautiously. Because atrophic skin is fragile and more susceptible to thermal injury, only low-energy, low-density settings are recommended to minimize the risk of necrosis [36]. AFR is typically indicated in the chronic phase, once the wound has fully epithelialized and scar maturation has plateaued, particularly when other modalities fail to restore skin integrity.

Hypertrophic Scars and Symptomatic Scarring

Hypertrophic scars, commonly seen in burn and trauma patients, are characterized by excessive fibroblast proliferation and disorganized collagen deposition driven by pro-fibrotic cytokines such as TGF-β and IGF-1 (Figure 3A) [44]. These raised, erythematous, and firm lesions impair skin compliance, limit socket tolerance, and frequently cause significant discomfort. Pain and pruritus, hallmarks of symptomatic scars, are also prevalent and interfere with prosthetic use and physical rehabilitation.

Laser therapy provides both functional and symptomatic relief [9]. For mature hypertrophic scars, AFR is the preferred modality due to its superior ability to remodel collagen and restore pliability [34]. In contrast, immature hypertrophic scars respond better to lower-density non-ablative fractional resurfacing (NAFR) or pulsed dye laser (PDL), which promote scar maturation without destabilizing the wound bed [36]. Treatment should be initiated after the inflammatory phase has resolved, often at three to six months post-injury, once the scar begins to exhibit signs of abnormal proliferation.

Both fractional and vascular lasers have demonstrated efficacy in reducing scar-associated pain and pruritus. Vascular lasers, such as PDL and Nd:YAG, are considered first-line treatments for symptomatic hypertrophic scars [27]. They target superficial vasculature and inflammatory mediators, leading to a reduction in erythema and symptom burden. AFR and laser-assisted corticosteroid delivery are effective second-line interventions, particularly for thick or treatment-resistant scars that have failed to respond to topical agents and conservative therapies. 

In the context of residual limbs, AFR has shown rapid and durable improvements in wound healing, pliability, and mechanical tolerance, sometimes within weeks, using straightforward post-procedural care such as vinegar soaks and petroleum jelly [9,45]. This is particularly valuable in managing scar contractures and dysfunctional scars that otherwise limit prosthetic engagement. Shumaker et al. recommend treating thicker scars with higher pulse energy, but the lowest available density to avoid exacerbation [40]. Scar maturity also plays a critical role, as immature scars (under 12-18 months) are more susceptible to laser-induced hypertrophy [36].

Laser-Assisted Drug Delivery (LADD)

LADD offers a promising adjunct to laser therapy in managing recalcitrant or symptomatic scars. The stratum corneum poses a significant barrier to topical medication absorption. Fractional lasers-both 2940 nm Er:YAG and 10,600 nm CO₂-create microscopic treatment zones (MTZs) that bypass this barrier, facilitating direct transdermal delivery of pharmacologic agents [46].

This technique has shown particular utility in enhancing corticosteroid penetration. In a porcine model, prednisone absorption increased proportionally with MTZ depth and density [47]. Clinically, AFR followed by triamcinolone application at two-to-three-month intervals has yielded up to 91% improvement in scar appearance [48]. However, increased drug delivery also raises the risk of corticosteroid-related adverse effects, such as dermal atrophy and telangiectasia [49].

LADD also supports the treatment of atrophic scars. Low-energy AFR followed by poly‐L‐lactic acid (PLLA) application promotes fibroblast activation and dermal thickening [36]. For hypertrophic scars, 5-fluorouracil (5-FU) is a viable alternative to corticosteroids. In a comparative trial, 5-FU and triamcinolone showed equivalent scar volume reduction, but only the steroid group exhibited adverse effects such as dermal thinning and telangiectasia [50,51].

While both ablative and non-ablative fractional lasers can facilitate drug delivery (with the former achieving a greater depth of penetration), the technique requires specialized equipment and provider expertise [52,53]. Cost may also limit accessibility [46]. Safety considerations, such as avoiding teratogenic agents like 5-FU in pregnancy, must also be observed.

Laser Hair Removal

Hair growth at the residual limb-prosthetic interface can cause significant mechanical irritation and friction under load. These conditions predispose patients to a variety of dermatologic complications, including bacterial folliculitis (Figure 3B), furunculosis, and irritant contact dermatitis, all of which may reduce compliance with prosthetic use and hinder rehabilitation efforts [7].

Mechanical shaving, often used to manage hair growth, is suboptimal and may exacerbate local irritation. It can lead to pseudofolliculitis barbae, microabrasions, and increased susceptibility to secondary infection [54]. Laser hair removal targets the pilosebaceous unit to selectively destroy the melanin chromophore within hair follicles. This depilation reduces friction and skin maceration, thereby improving dermal integrity at the prosthetic interface. Additional benefits include reduced heat retention and sweat accumulation, factors that contribute to skin breakdown and discomfort [9,55].

Laser hair removal has demonstrated marked improvements in health-related quality of life (HRQOL) among individuals with lower limb amputations. Miletta et al. evaluated 20 patients with residual limbs treated with two to six laser hair removal sessions and found that 90-100% of participants reported improvements in symptoms, emotional well-being, functional ability, and overall HRQOL within six weeks post-treatment, as measured by the Skindex-16 dermatologic quality-of-life instrument [54]. Clinically, patients experienced significant reductions in ingrown hairs, prosthetic slippage, hyperhidrosis, and limitations in physical activity.

Given these findings, laser hair removal should be considered as a prophylactic or adjunctive intervention in the early post-prosthetic fitting phase or in cases where recurrent folliculitis or persistent friction-related dermatoses are present. Dermatologists or trained specialists can tailor treatment parameters based on individual skin phototypes. For example, Koch et al. demonstrated efficacy using a 755 nm Alexandrite laser for Fitzpatrick skin types II-IV and a long-pulsed 1064 nm Nd:YAG laser for types IV-V, with treatments administered every six to eight weeks over 2-11 sessions [56]. Over a three-year follow-up period, treated patients experienced sustained clinical benefit, minimal adverse effects, and high satisfaction rates.

In clinical experience, laser hair removal is uncomfortable but generally better tolerated than ablative fractional resurfacing lasers. Patients with amputations often present with heterogeneous patterns of sensation across the residual limb; some areas demonstrate hyperesthesia, while others lack all feeling entirely. In most cases, the application of a topical anesthetic in a thick layer under occlusion approximately one hour prior to treatment provides sufficient analgesia. Areas that remain highly sensitive can typically be avoided during treatment, unless the hair growth in those regions is clearly contributing to pathology, such as chronic folliculitis or ulceration. In those cases, localized injectable anesthesia can be used to enable adequate treatment while minimizing patient discomfort. Patients and providers alike should be aware that this procedure is not necessarily permanent, as response to treatment varies among individuals, and it may take 6-12 sessions to achieve meaningful outcomes [9,54]. Permanent hair reduction is more likely if an area remains hairless for at least one year following treatment [57]. The reduction in sweating, thought to result from non-selective thermal injury to eccrine glands, is usually transient and may require periodic maintenance treatments [54]. Importantly, laser hair removal is ineffective on red, light blonde, or white hair due to insufficient melanin chromophore content.

Epidermal Inclusion Cysts

EICs form when epidermal components become entrapped within the dermis, leading to the accumulation of keratinous material within a cystic cavity. On residual limbs, EICs commonly arise from chronic shearing forces, wherein repetitive pressure and friction from prosthetic use cause keratin invagination around the hair follicle [12,58]. These lesions can be painful, prone to secondary infection, and may interfere with prosthetic fit and comfort, ultimately limiting functional mobility.

Surgical excision remains the standard treatment for EICs; however, excision carries the risk of hypertrophic or keloid scar formation, particularly in high-tension or previously compromised skin regions. In the context of the residual limb, where optimal skin integrity is critical for prosthetic compatibility, minimally invasive alternatives may be desirable. Laser therapy has emerged as a promising treatment modality for small EICs, offering the advantage of precise tissue ablation with reduced trauma to surrounding structures [59].

Multiple laser platforms, including CO₂, Nd:YAG, and, more recently, Er:YAG lasers, have demonstrated efficacy in the treatment of EICs. In a case series of 25 patients undergoing Er:YAG laser fenestration of non-infected EICs, 92% achieved effective resolution without recurrence, and notably, no cases of hypertrophic scarring were observed [58]. This approach may be particularly beneficial in patients with residual limbs, where preserving skin pliability and minimizing scar formation are paramount for maintaining prosthetic tolerance and preventing future complications. This technique is best suited for small to moderately sized, non-infected EICs and can be considered in patients with a history of poor wound healing or when surgical excision may pose undue risk to prosthetic function.

Psoriasis

Psoriasis is a chronic autoimmune inflammatory skin disorder that may develop or worsen on residual limbs due to mechanical trauma and repetitive irritation from prosthetic use. This trauma can trigger immune activation and lesion development through a phenomenon known as Koebnerization [7]. On the residual limb, psoriatic plaques may become symptomatic and interfere with prosthetic wear, particularly if located in weight-bearing or high-friction regions.

The excimer laser, which emits monochromatic ultraviolet B (UVB) light at 308 nm, is currently used in the treatment of localized plaque psoriasis and is FDA-approved for this indication. The laser induces apoptosis in hyperproliferative keratinocytes and pathogenic T lymphocytes, thereby suppressing the local immune response that drives disease activity [60]. Excimer laser therapy is well-tolerated, with minimal adverse effects such as transient erythema or blistering, and can be used in conjunction with topical medications [61]. Clinically, it is most useful for treating plaques that are refractory to topical agents or systemic immunosuppressants, especially when localized to areas that affect prosthetic fit and function.

While topical anti-inflammatory agents, particularly corticosteroids, remain the first-line therapy for limited focal plaques, they carry the risk of side effects such as skin atrophy, telangiectasia, and tachyphylaxis with long-term use. In select cases, particularly on the residual limb where lesions may arise from Koebnerization and require repeated management, excimer laser treatment can offer a more targeted and elegant solution. Although multiple treatment sessions are typically required, which may be viewed as a limitation, laser therapy avoids the cumulative adverse effects associated with chronic topical corticosteroid use [62]. As such, laser therapy may be preferred for persistent or anatomically sensitive plaques where minimizing local tissue compromise is paramount for prosthetic tolerance and long-term skin health.

Complications of laser treatment

Complications associated with laser therapy primarily arise from unintended or non-selective thermal injury to surrounding tissue. The most commonly reported adverse effects include blistering, crusting, dyspigmentation, and, less frequently, scarring. In the context of laser hair removal, complications are rare but may occur due to the unintended absorption of laser energy by epidermal melanin during transmission into the deeper hair follicle target [63]. This risk is particularly elevated in individuals with darker skin types (Fitzpatrick IV-VI), whether due to constitutive pigmentation or increased ultraviolet (UV) exposure, owing to the higher concentration of melanin in the epidermis [64].

**Figure 4:**
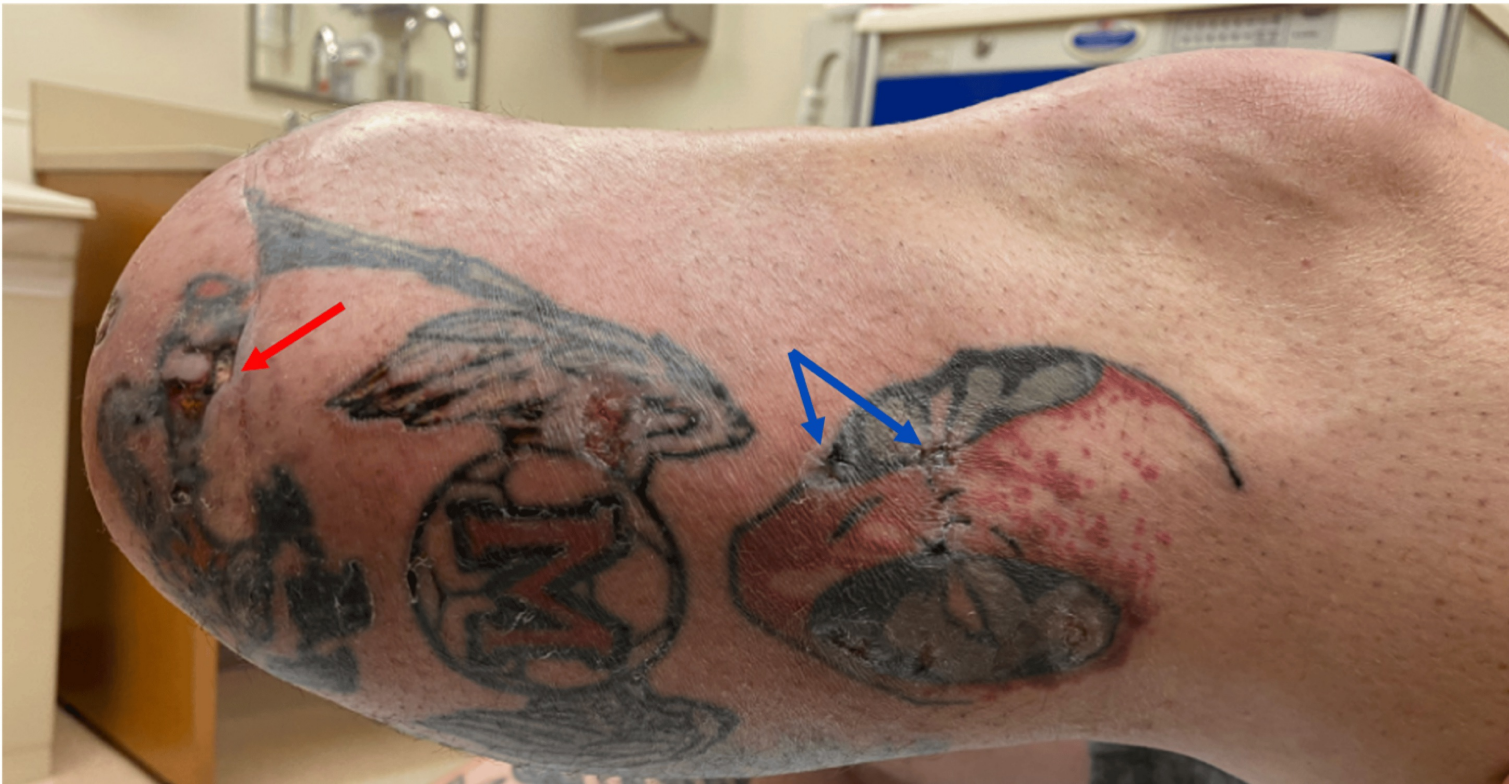
Complication from laser hair removal on residual limb Patient with complications from the Alexandrite laser used for laser hair removal. It is to be noted that areas treated containing tattoo pigment resulted in blisters (blue arrows), and in one area, a full-thickness ulceration (red arrow). Image Source: Authors; The patient provided written informed consent for the use of this image in this open-access publication.

A critical and absolute contraindication to laser hair removal is treatment over areas of tattooed skin. Similar to melanin, tattoo pigments can absorb laser energy, regardless of wavelength, resulting in excessive thermal buildup. Even with wavelengths such as 1064 nm (Nd:YAG), which are generally considered safer for deeper targeting and darker skin tones, pigment within tattoos may still absorb significant energy. This can result in localized epidermal damage, blistering, necrosis, and potentially permanent scarring (Figure 4). Consequently, laser hair removal should never be performed over tattooed regions of the residual limb.

To reduce the risk of adverse outcomes more broadly, laser parameters must be carefully selected to limit thermal diffusion to adjacent non-target tissue. This includes the use of longer wavelengths, extended pulse durations, and larger spot sizes. These adjustments enhance penetration depth and treatment efficiency while decreasing the required fluence, thereby minimizing collateral thermal injury [64-=]. For example, the 1064 nm Nd:YAG laser, with its longer wavelength, is more selectively absorbed by deeper structures and demonstrates reduced epidermal absorption, making it more suitable for darker skin types. In contrast, lasers with shorter wavelengths, such as the 755 nm Alexandrite laser, are better suited for individuals with lighter skin tones (Fitzpatrick I-III) [59]. Epidermal cooling is another critical strategy to minimize thermal injury and can be administered prior to (pre-cooling), during (parallel cooling), or after treatment (post-cooling), depending on the device and clinical setting [23].

Data on complications following non-ablative fractional resurfacing (NAFR) and ablative fractional resurfacing (AFR) for scar treatment remain limited, though the available evidence suggests a favorable safety profile [29,36]. Common side effects include transient erythema and edema, typically resolving within several days. In the case of AFR, however, these effects may persist for up to six weeks [23,36]. Immediate post-treatment application of topical corticosteroids has been shown to reduce the duration and intensity of this inflammatory response [23]. Pinpoint bleeding is also observed, particularly with Er:YAG laser treatment, and is generally less frequent with CO₂-based systems [36]. Infection is a potential complication following fractional laser procedures, but it occurs infrequently. Routine prophylactic antibiotic administration before or after treatment is no longer standard practice and is instead guided by patient-specific risk factors and clinical judgment [29].

## Conclusions

Laser therapy offers a minimally invasive and versatile alternative to conventional treatments for dermatologic complications on the residual limb. By effectively managing conditions like contractile scars, follicular disorders, and inclusion cysts, lasers can enhance prosthetic use and increase patient function. When applied with appropriate clinical judgment, laser treatment is a safe and targeted approach that can significantly improve skin integrity. Integrating these therapies into multidisciplinary care is crucial for advancing both dermatologic and rehabilitative outcomes for individuals with limb loss. 
